# Uptake of heavy metal ions from aqueous media by hydrogels and their conversion to nanoparticles for generation of a catalyst system: two-fold application study†

**DOI:** 10.1039/c8ra00578h

**Published:** 2018-04-18

**Authors:** Rida Javed, Luqman Ali Shah, Murtaza Sayed, Muhammad Saleem Khan

**Affiliations:** National Centre of Excellence in Physical Chemistry, University of Peshawar Peshawar 25120 Pakistan luqman_alisha@yahoo.com Luqman_alisha@uop.edu.pk +92 919216671 +92 3455707518

## Abstract

Poly(methacrylic acid) (P(MAA)), poly(acrylamide) (P(AAm)) and poly(3-acrylamidopropyltrimethyl ammonium chloride) (P(APTMACl)) were synthesized as anionic, neutral and cationic hydrogels, respectively. The synthesized hydrogels have the ability to be used as absorbents for the removal of selected heavy metal ions such as Cu^2+^, Co^2+^, Ni^2+^ and Zn^2+^ from aqueous media. Absorption studies revealed that the absorption of metal ions by the hydrogels followed the order Cu^2+^ > Ni^2+^ > Co^2+^ > Zn^2+^. For the mechanism of absorption, both Freundlich and Langmuir absorption isotherms were applied. Metal ion entrapped hydrogels were treated using an *in situ* chemical reduction method in order to convert the metal ions into metal nanoparticles for the synthesis of hybrid hydrogels. The synthesis and morphology were confirmed using FT-IR and SEM, while the absorbed metal amounts were measured using TGA and AAS. The hybrid hydrogels were further used as catalysts for the reduction of macro (methylene blue, methyl orange and congo red) and micro (4-nitrophenol and nitrobenzene) pollutants from the aqueous environment. The catalytic performance and re-usability of the hybrid hydrogels were successfully investigated.

## Abstract 2

Poly(methacrylic acid) (P(MAA)), poly(acrylamide) (P(AAm)) and poly(3-acrylamidopropyltrimethyl ammonium chloride) (P(APTMACl)) were synthesized as anionic, neutral and cationic hydrogels respectively.

## Graphical abstract

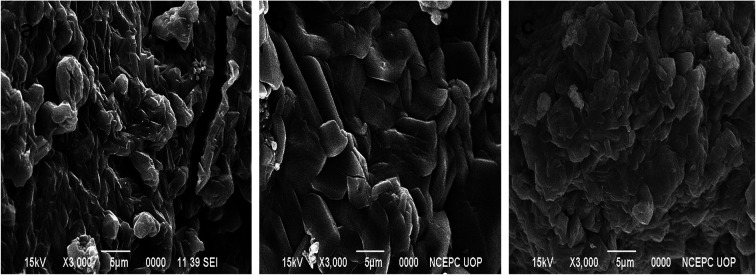

## Introduction

Addressing water pollution is an immense task for researchers, as over the past few years, it has become more and more of an issue due to manufacturing effluents, which are directly added to seas, rivers, lakes, oceans, *etc.* without initial treatment. The major causes of water pollution are inorganic heavy metal ions and organic macro (dyes) and micro (nitroarenes) pollutants. The major sources of these pollutants are effluents from the textile, pharmaceutical, food additive, cleaning, dyeing and leather industries, which are added to bodies of water.^1^ The exposure of these pollutants to the human body and to other animals (both terrestrial and aquatic) causes severe health problems, *e.g.* a surplus amount of heavy metal ions causes anaemia, diarrhoea, nausea, abdominal pains, reduction of red blood cells, narrowing of muscles, hypoglycaemia or even death.^2–5^ Similarly, the existence of organic pollutants like dyes, nitroarenes, *etc.* in bodies of water has become a vital issue everywhere but more specifically in the developing world due to their high toxicity, low degradability and persistent bioaccumulation. Dyes are organic compounds used in industries for multiple purposes because of their cheapness and high availability, but upon being released in water they cause many problems in aquatic as well as in terrestrial living organisms, both directly and indirectly,^6^*e.g.* methylene blue (MB) results in heart problems, nausea, jaundice, tissue rupture, trembling and Heinz body formation in humans.^7^ Congo red is extremely stable and resists degradation, so it is lethal for living organisms and is a cause of cancer in humans^8^ due to the aggregation of platelets that leads to allergies and sometimes metabolites that produce benzidine, which causes bladder cancer in humans.^9^ Nitroaromatic compounds cause damage to the brain, liver and kidney, affect the circulatory system of living organisms, *etc.*

These inorganic (heavy metals) and organic (micro and macro) pollutants badly affect the lives of humans and animals, as well as plants. Elimination of these pollutants from wastewater to make it suitable for usage is a thought-provoking task for researchers.^10^

Until now, various physical, chemical, and biological techniques have been used by different researchers for the elimination of pollutants, such as precipitation,^11^ ion exchange,^12^ adsorption,^13^ ultrafiltration,^14^ coagulation and flocculation,^15^ catalysis,^16–19^ degradation,^20–23^*etc.* These treatment processes are often not easy to maintain, not efficient and very costly.

In recent years, there have been numerous hydrogel systems used for the removal of heavy metal ions and organic pollutants that showed good absorbance activity, as they have porous three dimensional cross-linked structures with hydrophilic groups that can hold the maximum amount of water in their matrix.^24,25^ Hydrogels have extraordinary properties, such as simple preparation, cost effectiveness, high efficiency, easy separation and good stability, and they can be reused for a number of cycles.^26^

The hydrogels used so far are only for one purpose, but in this paper we have highlighted a two-fold application of hydrogels. Firstly, the hydrogels were used as uptake sources for selected heavy metal ions from wastewater; secondly, the entrapped metal ions were reduced to metal particles *via in situ* reduction for the generation of hybrid hydrogel catalyst systems, which were further applied as catalysts for the reduction of organic pollutants. We have prepared anionic poly(methacrylic acid) P(MAA), neutral poly(acrylamide) P(AAm) and cationic poly(3-acrylamidopropyltrimethyl ammonium chloride) P(APTMACl) hydrogels *via* a free radical polymerization process. The uptake capacity of the prepared hydrogels was examined against selected heavy metal ions *i.e.* Cu, Co, Ni and Zn. The entrapped heavy metal ions inside the hydrogels were reduced to metal nanoparticles *via* an *in situ* chemical reduction method, using NaBH_4_ as the reducing agent. Hybrid hydrogels were used as catalysts for the reduction of macro (MB, MO and CR) and micro (4-NP and NB) pollutants. The stability of the hybrid hydrogels was good and they can be recycled up to 5 times with negligible loss in catalytic efficiency. Moreover, this is a simple, convenient, cost effective, efficient and reliable method for the removal of inorganic and organic pollutants from wastewater.

## Experimental

### Materials

Methacrylic acid (MAA, 99.5%; ACROS), acrylamide (AAm; ORGANICS) and (3-acrylamidopropyl) trimethyl ammonium chloride (APTMACl, 75% wt in water; Sigma Aldrich) were used as anionic, neutral and cationic monomers, respectively. *N*,*N*-Methylene bis acrylamide (MBA, 99%; Sigma Aldrich) was used as a cross linker, ammonium persulfate (APS, 98%; Sigma Aldrich) as an initiator, and *N*,*N*,*N*′,*N*′-tetramethylethylenediamine (TEMED, 99% pure) was used as an accelerator. All of these chemicals were used for the synthesis of hydrogels as received. Copper(ii) chloride hexahydrate (CuCl_2_·6H_2_O), cobalt(ii) chloride hexahydrate (CoCl_2_·6H_2_O), nickel(ii) chloride hexahydrate (NiCl_2_·6H_2_O) and zinc(ii) nitrate hexahydrate (Zn(NO_3_)_2_·6H_2_O) were purchased from KOSDAQ and used as metal ion sources, while sodium borohydride (NaBH_4_ 98%; Sigma Aldrich) was used as a reducing agent. Congo red (CR, Sigma Aldrich), methylene blue (MB, Sigma Aldrich) and methyl orange (MO, BDH) as sources of macro-pollutants and 4-nitrophenol (4-NP) and nitrobenzene (NB) from Sigma Aldrich as sources of micro-pollutants were used. Milli-Q distilled water was used throughout the experimental work.

### Synthesis of pure hydrogels

Poly(MAA), poly(APTMACl) and poly(AAm) based hydrogels were prepared *via* free radical polymerization at room temperature. 0.5 g of each monomer was taken and poured into three vials each containing 4 mL of water, and after that 0.1 g of BIS and 50 μL TEMED were added to each vial. After the addition of TEMED, each mixture was vortexed until a homogeneous solution was attained. APS solution (0.05g mL^−1^ in H_2_O) was added to each vial to start polymerization, and the corresponding polymer hydrogels were formed within 5 min, as shown in Fig. S1.† The materials were washed of impurities and other unreacted monomers by keeping them in DI water for 24 hours. After washing, the hydrogels were dried in an oven at 50 °C (inset of Fig. S1†) and stored for further use.

### Absorption study

The prepared hydrogels were used as absorbents for the removal of heavy metal ions from solution. For this purpose, solutions of selected heavy metal ions (*i.e.* Co^2+^, Cu^2+^, Ni^2+^ and Zn^2+^) of different concentrations (*i.e.* 5 ppm, 10 ppm, 20 ppm, and 40 ppm) were prepared and the maximum absorption limit of the hydrogels towards the heavy metal ions was calculated. In each batch of experiments, 0.5 g of each hydrogel sample was cut into pieces before putting the pieces into 20 mL salt solutions at room temperature, which were stirred at 250 rpm for 12 h. After stirring, the hydrogels were separated simply *via* centrifugation. The concentration of the metal ions was checked in the separated solution using flame atomic absorption spectroscopy (AAS) (Perkin Elmer AAnalyst 800 atomic absorption spectrophotometer with a graphite furnace atomizer, Zeeman background correction and an autosampler) and the absorption capacity was found for the prepared hydrogels.

### Synthesis of hybrid hydrogels

The entrapped metal ions in the P(MAA), P(APTMACl) and P(AAm) hydrogels were reduced to metal particles by NaBH_4_ (0.15 g per 10 mL of H_2_O) for the synthesis of the hybrid gels. The formation was done in a three necked round bottom flask for 30 min under an inert atmosphere until the bubbles of H_2_ stopped. When hybrid hydrogels were formed, a prominent change in color was observed. Cu, Co and Ni containing hydrogels appeared black in colour, while Zn gives a white colour, as illustrated in Fig. S2.† The synthesized materials were purified, dried at 60 °C and converted to powder form by grinding with a pestle and mortar for further use.

### Catalytic and recycling performance

The catalytic and recycling performance of hybrid gels were checked in the reduction process of macro-pollutants like organic dyes (MB, MO and CR) and micro-pollutants such as 4-NP and NB with different concentrations using NaBH_4_. The catalytic reduction process was monitored using UV-visible spectroscopy. To find out the catalytic activity of the synthesized catalysts (hybrid hydrogels), solutions of the dyes MO, MB and CR with 17 ppm, 8 ppm and 22 ppm, respectively, and nitroarene (4-NP and NB) solutions with a concentration of 0.01 M were prepared. For each process, 3 mL of solution was put into a quartz cell followed by a small amount of the catalyst along with an excess amount of the reducing agent, and the reduction in *λ*_max_ for the pollutants was recorded at different time intervals. The catalysts were recycled using centrifugation, washed with pure water and employed continuously for five consecutive cycles.

### Characterization

The surface morphologies of the synthesized materials were visualized using SEM (JSM-5910, JEOL). The FT-IR spectra were recorded for functional group and incorporated metal determination using a Shimadzu IR Prestige-21 spectrophotometer. The thermal stability and percent content of the metals were calculated using TGA (Diamond TGA/DTA Perkin Elmer). Flame atomic absorption spectroscopy (AAS) (Perkin Elmer AAnalyst 800 atomic absorption spectrophotometer with a graphite furnace atomizer, Zeeman background correction and an autosampler) was applied for the determination of the metal ions taken by the polymer hydrogels from the aqueous medium. A UV-visible spectrophotometer (Perkin Elmer) was used to control the reduction process for the organic pollutants (dyes and nitroarenes).

## Results and discussion

### Synthesis of pure and hybrid hydrogels

P(MAA), P(AAm) and P(APTMACl) were synthesized *via* free radical polymerization. The process of polymerization was initiated and accelerated by APS and TEMED, respectively, and a three dimensional network of the hydrogel was achieved due to the presence of the cross linker BIS in the reaction mixture. Both P(MAA) and P(APTMACl) are ionic in nature and they change their behavior with the pH of the surrounding medium, while P(AAm) is neutral with homogeneous properties at any environmental pH value. In P(MAA), the entrapment of metal ions was due to the electrostatic force of attraction and diffusion, while in P(AAm) and P(APTMACl), the penetration of metal ions was only through a diffusion process. Due to this behavior, the absorption capacity of P(MAA) was greater than that of P(AAm) and P(APTMACl) for metal ions from aqueous solution. All three hydrogels, P(MAA), P(AAm) and P(APTMACl), were used as templates for the absorption of selected heavy metal ions (*i.e.* Cu, Co, Ni and Zn). These metal ions were reduced to their atomic forms for the generation of nanoparticle entrapped hybrid hydrogels.

**Fig. 1:**
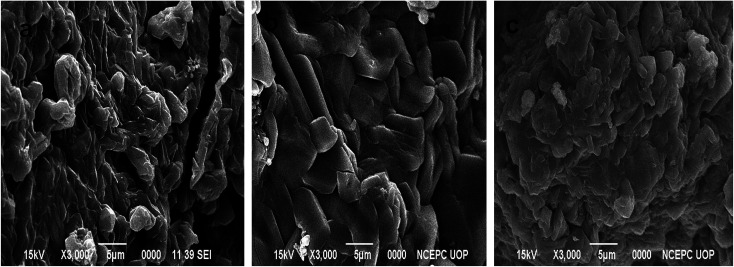
SEM images of prepared pure (a) P(MAA) (b) P(APTMACl) and (c) P(AAm) hydrogels.

**Fig. 2:**
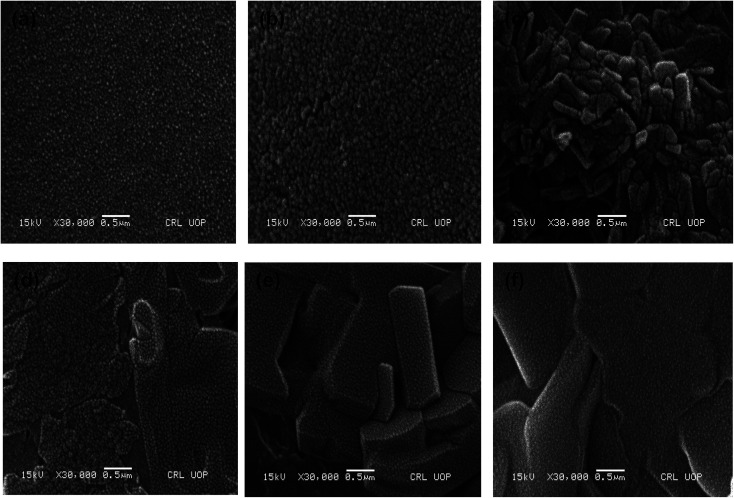
SEM images of hybrid (a) P(MAA)–Cu (b) P(MAA)–Ni (c) P(APTMACl)–Cu (d) P(APTMACl)–Ni (e) P(AAm)–Cu and (f) P(AAm)–Ni hydrogels.

The SEM micrographs for the pure P(MAA), P(AAm) and P(APTMACl) hydrogels showed that the P(MAA) surface was more porous and rough than that of P(AAm) and P(APTMACl), as shown in Fig. 1. Generally, a material with a rougher surface will have a higher absorption capacity. In this study, P(MAA) showed the highest absorption capacity due to its rough nature. SEM images of the hybrid hydrogels showed that metal nanoparticles are distributed into the matrix without any aggregation, as illustrated in Fig. 2(a–f). The existence of metal was further confirmed using EDX analysis and the results are given in Fig. S3.† This shows that hydrogels that were used as a stabilizer or uptaker for nanoparticles proved to be the best support. In P(MAA), P(AAm) and P(APTMACl), encapsulated nanoparticles showed long term stability inside the network, because of the interaction between the functional groups of the hydrogels and the nanoparticles. The carboxyl group of methacrylic acid and the amide groups of acrylamide and 3-acrylamidopropyltrimethyl ammonium chloride donate electrons to the metal nanoparticles. This interaction due to the donation and acceptance of electrons gives stability to the metal nanoparticles. This type of coordination interaction of metal particles and hydrogels was also reported by Chen *et al.*^27^

**Fig. 3:**
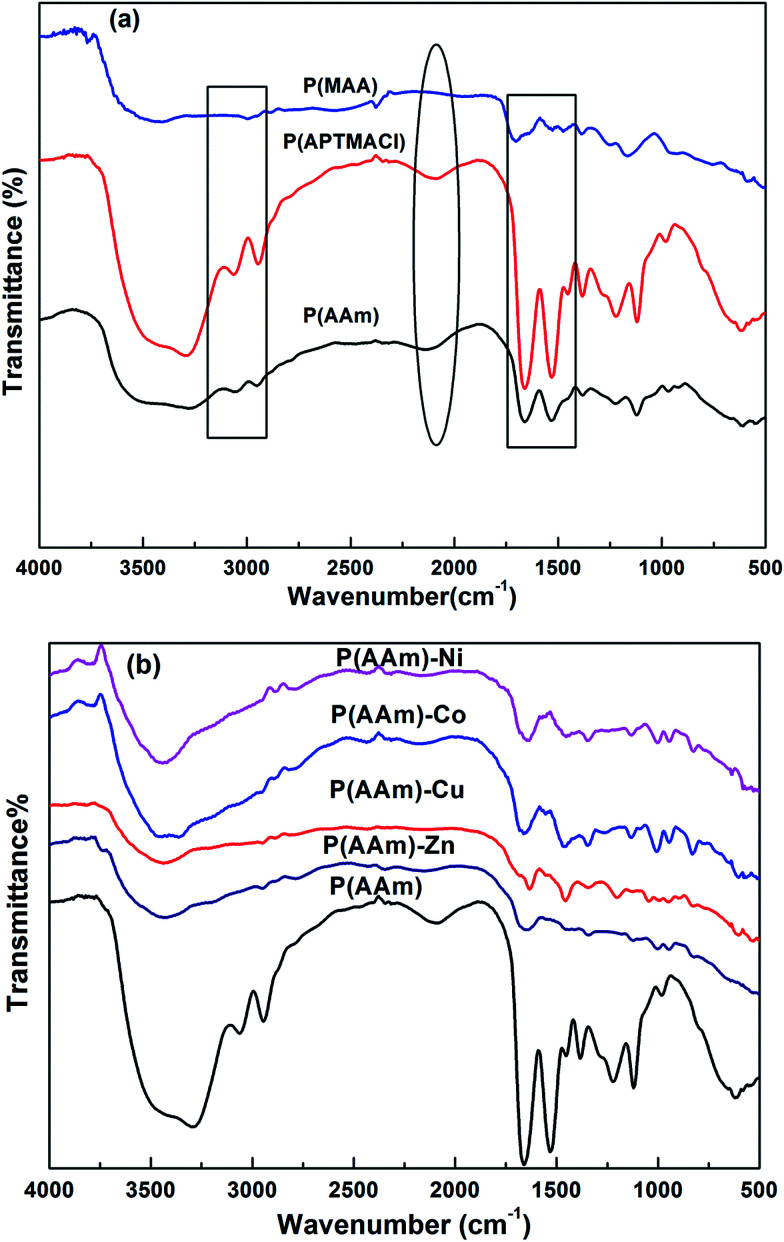
FT-IR spectra of (a) pure hydrogels and (b) P(AAm) pure and hybrid hydrogels.

The FT-IR spectra of synthesized P(MAA), P(AAm) and P(APTMACl) are given in Fig. 3a. In the FT-IR spectrum for the P(MAA) hydrogel, a wide stretching vibration band of an –OH group appeared at 3280 cm^−1^, while no such band was observed for P(APTMACl) and P(AAm) hydrogels. Similarly, the band for C=O for P(MAA) was observed at 1701 cm^−1^, while for P(AAm) and P(APTMACl), the C=O peak was shifted to 1660 cm^−1^. This shift was due to the attachment of an amide group, which shows more resonance ability than the –OH group of P(MAA). Similarly, the –CH symmetric and asymmetric stretching bands for the P(MAA), P(APTMACl) and P(AAm) hydrogels were observed at 2994 cm^−1^, 3054 and 2948 cm^−1^, and the intensity of the peaks at 3054 and 2934 cm^−1^ was highest for P(APTMACl), lowest for P(MAA), and in between these for P(AAm). The NH bending band of the amide group for the P(APTMACl) and P(AAm) hydrogels was observed at 1532 cm^−1^, and no such band appeared for P(MAA).^28^ These results indicate the successful synthesis of polymer hydrogels.

FT-IR spectra of the pure and hybrid hydrogels of P(AAm) are given in Fig. 3b. The presence of metal nanoparticles produces a shift in the peaks corresponding to the groups involved in interaction with the metal nanoparticles. For all of the P(MAA) hybrid hydrogels, the –OH stretching band shifts from 3423 cm^−1^ for the pure P(MAA) hydrogel to 3416 cm^−1^ for all hybrid hydrogels, and the C=O band shifts from 1701 cm^−1^ for the pure hydrogel to 1641, 1641, 1634 and 1647 cm^−1^ for Co, Cu, Ni and Zn hybrid hydrogels, respectively.

In P(AAm) nanoparticle hybrid hydrogels, the stretching band of –NH shifted from 3440 to 3430 cm^−1^, the –C=O band shifted from 1654 cm^−1^ to 1627, 1647, and 1647 cm^−1^ and the –C–N band shifted from 1453 to 1460, 1453, 1440 and 1433 cm^−1^ for Co, Cu, Ni and Zn hybrid hydrogels. Similar results were observed for P(APTMACl) but are not shown here.

**Fig. 4:**
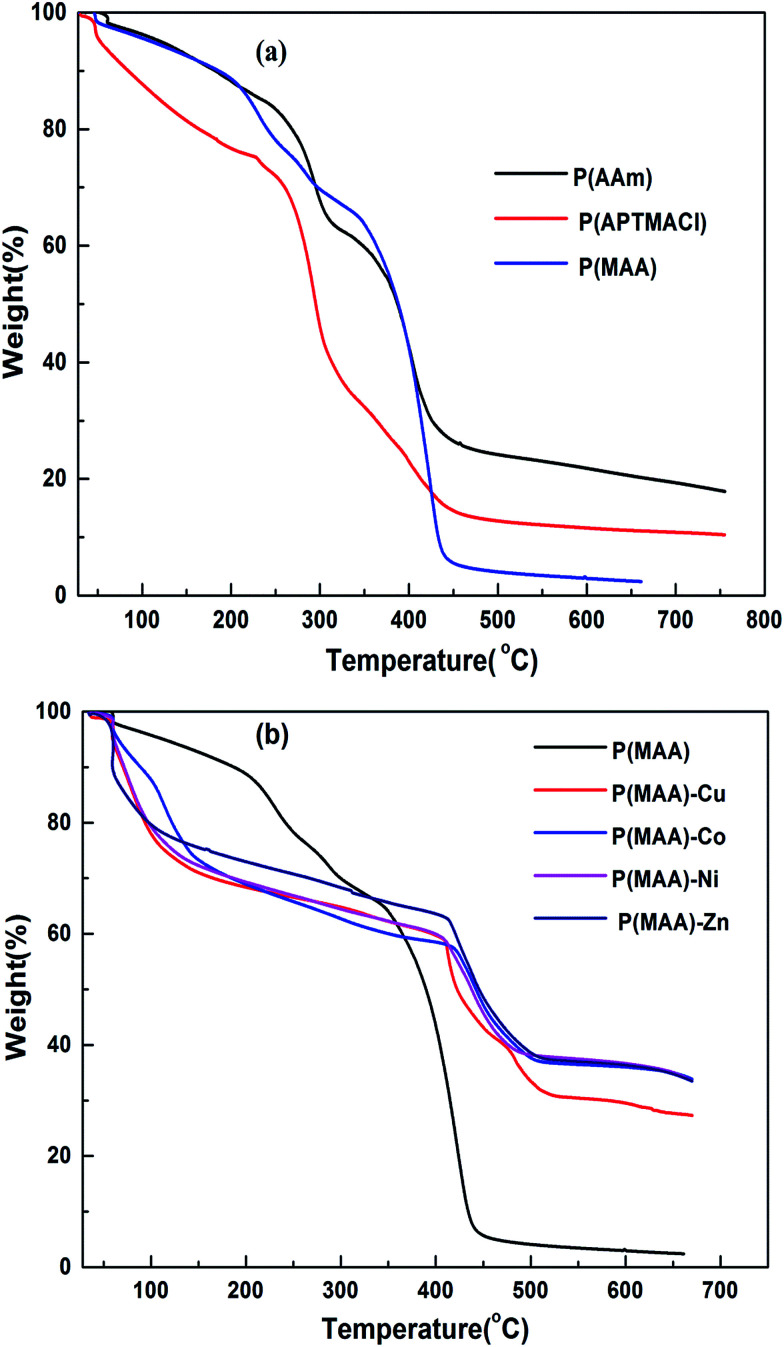
TGA curves for (a) pure hydrogels and (b) P(MAA) pure and metal entrapped hybrid hydrogels.

Thermal gravimetric analysis (TGA) was carried out to determine the metal content absorbed by the hydrogels by measuring the thermal degradation behaviour of pure and hybrid hydrogels. Thermograms for the pure and hybrid hydrogels are shown in Fig. 4(a) and (b), respectively, with a heating range from room temperature up to 700 °C. It is significant to state here that pure hydrogels have two distinct degradation transitions associated with different temperature and % weight loss. The results indicate 33 and 60 wt% loss for P(MAA) at 58 and 343 °C, similarly 21 and 61 wt% loss for P(APTMACl) at 50 and 230 °C, and 34 and 35 wt% loss for P(AAm) hydrogels at 70 and 336 °C.

Therefore, the total weight lost at 650 °C for P(MAA), P(APTMACl) and P(AAm) hydrogels were 93, 82 and 69%, respectively. This indicates that P(MAA) has a higher water content inside the network structure compared to the P(APTMACl) and P(AAm) hydrogels.

After incorporation of metal nanoparticles, the thermal behaviour was totally changed, and an increase in the thermal stability was observed due to entanglement and entrapment of complexed nanoparticles with polymer chains.^29^ The TGA results give thermal stability and not exactly, but almost exactly, the % amount of metal particles absorbed by the hydrogels. The exact amount cannot be calculated because of the formation of some metal oxides due to the abundant existence of the –COOH group within the hydrogel. The thermal degradation for pure P(MAA) and hybrid hydrogels is described in Table S1,† which shows that the metal content absorbed by P(MAA)–Cu and P(MAA)–Zn was 26%, and for P(MAA)–Ni and P(MAA)–Co it was 25% at 650 °C. Similarly, the thermal decomposition results for the P(AAm) and P(APTMACl) hybrid hydrogels are presented in Table S2,† which shows that a lower metal content was absorbed by P(AAm) and P(APTMACl) compared to the P(MAA) hydrogels. This is in accordance with the SEM results, which indicated that the synthesized P(MAA) hydrogel was more rough and porous and thus had a high capacity for metal ions.

### Uptake capacity of hydrogels

The uptake capacities of the P(MAA), P(AAm) and P(APTMACl) hydrogels were examined for the removal of selected heavy metal ions *i.e.* Cu^2+^, Co^2+^, Ni^2+^ and Zn^2+^ from the corresponding aqueous medium by measuring the concentration of the metal ions before and after their interaction with the hydrogels.

Salt solutions with different concentrations ranging from 1–40 ppm were prepared in de-ionized water. 20 mL of each salt solution was loaded with a typical amount of the hydrogel and stirred for 12 h. After stirring, hydrogels with entrapped metal ions were separated from the salt solution by centrifugation, and the concentration of the salt ions before and after uptake was determined using an Atomic Absorption spectrometer.

From the unit mass of the hydrogels, the amount of metal uptake (mg g^−1^) and removal percentage were calculated using eqn (1) and (2), respectively:1
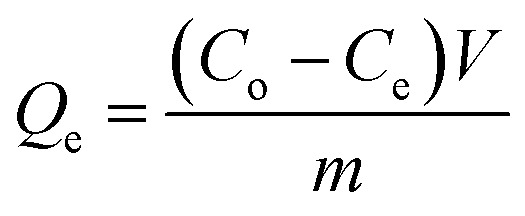
2
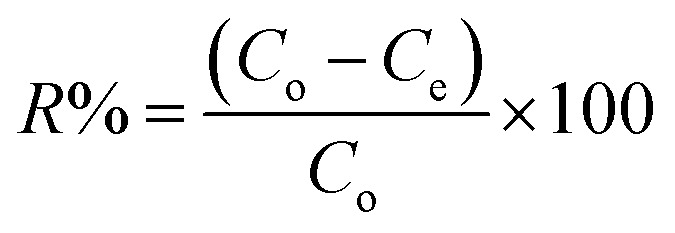
where *Q*_e_ is the amount of entrapped metal ions in mg g^−1^, *C*_o_ and *C*_e_ are the initial and equilibrium concentrations of metal ions in solution, *V* is the volume of the solution in litres and *m* is the mass of hydrogels used for the experiment.

The amounts of the metal ions calculated for different concentrations are illustrated in Table 1, which shows that the order of metal ions uptaken by hydrogels was Cu > Ni > Co > Zn, while the uptake capacity of the hydrogels follows the order P(MAA) > P(AAm) > P(APTMACl). The highest uptake capability for P(MAA) is because of the diffusion and electrostatic attractive forces between the carboxyl (COO–) groups and metal ions, which favors the penetration of metal ions inside the hydrogel network, while in P(AAm) the penetration of metal ions is due to the diffusion process only. The lowest uptake ability for P(APTMACl) is because of the electrostatic repulsive forces between the positive charge (NH_4_^+^) of the polymer chains and metal ions, which restrict its penetration into the hydrogel network, but still a lower amount of metal ions enters due to diffusion.

**Table 1:** Uptake capacity of hydrogels for selected heavy metal ions

Salt conc. (ppm)	P(MAA) uptake capacity (mg g^−1^)				P(AAm) uptake capacity (mg g^−1^)				P(APTMACl) uptake capacity (mg g^−1^)			
	Cu	Co	Ni	Zn	Cu	Co	Ni	Zn	Cu	Co	Ni	Zn
5	2.00	1.880	1.970	1.390	1.170	1.690	1.840	1.300	1.150	1.480	1.866	1.357
10	3.95	3.840	3.880	3.480	2.340	3.270	3.620	2.780	2.310	3.100	3.520	2.750
20	12.40	10.79	11.32	8.320	5.800	9.870	13.32	8.240	4.030	8.190	8.940	6.240
40	20.68	19.89	20.10	15.59	10.47	17.60	19.01	6.480	8.590	13.00	16.16	11.10

**Fig. 5:**
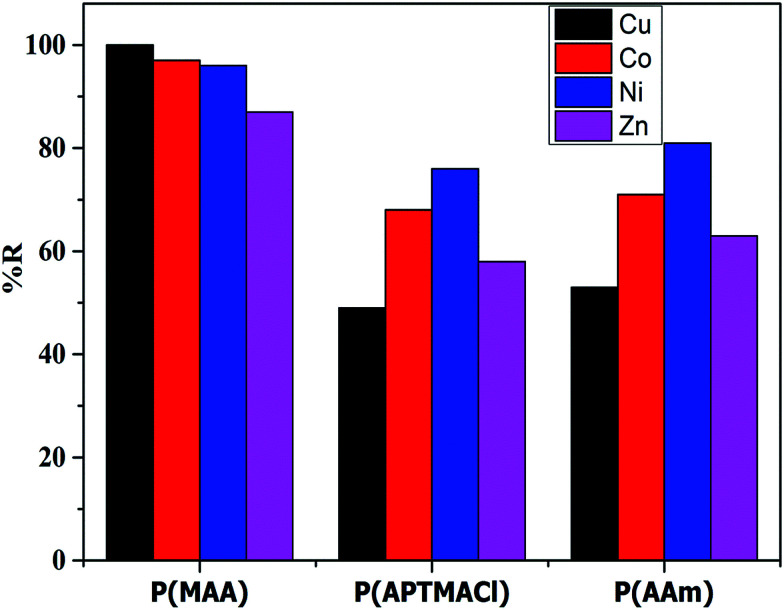
% *R* of hydrogels from 10 ppm salt solutions.

The removal percentages of the hydrogels for different salt concentrations were calculated and are tabulated in Table S3.† The results indicated that, in term of the hydrogels, the P(MAA) hydrogel shows a high and constant % *R* for different salt concentrations, whereas the P(AAm) and P(APTMACl) hydrogels show a decrease in % *R* for high salt concentrations. These results confirm the superior penetration capacity of P(MAA) due to electrostatic attraction and diffusion, while the penetration of metal ions through only diffusion in P(AAm), and in P(APTMACl) due to diffusion as well as electrostatic repulsion, reduces the available sites inside the hydrogels and decreases the removal capacity. In terms of the metal ions, the % *R* of the hydrogels for 10 ppm is plotted in Fig. 5, which shows the high capacity of P(MAA) for Cu^2+^, and similarly of P(APTMACl) and P(AAm) for Ni^2+^ and Co^2+^. As the charges of all of the metal ions are the same, the increase or decrease of their absorption by the hydrogels depends upon the size and complexation ability. For P(MAA), both electrostatic and diffusion processes are involved, so the amount of metal ions was the maximum, but for the P(APTMACl) hydrogel, there is only diffusion that is responsible for absorption as this hydrogel also faces electrostatic repulsion due to the same charge, which lowers its absorption capacity compared to that of P(AAm). Metal ions such as Ni^2+^ and Co^2+^, with a size of 70 pm, diffuse more rapidly than the other metal ions Cu^2+^ and Zn^2+^, with sizes of 73 and 74 pm, so a similarly rapid uptake capacity was shown by P(AAm) towards Ni^2+^ and Co^2+^.

At lower concentrations of metal ion solutions, a maximum value of % *R* was obtained; this is due to the availability of active groups and space inside the hydrogels for the accommodation of metal ions. However, when the whole available space is filled, then a further increase in the concentration of metal ions cannot be removed by the hydrogels.^30^ It will be possible only when we increase the amount of hydrogels by creating more active sites and space for the metal ions in the polymer hydrogels.

To find out the effect on the uptake efficiency of the hydrogels toward metal ions in the presence of other metal ions, competitive absorption experiments were performed by taking 20 mL of solution composed of 5 mL of each metal salt solution. To this solution, certain amounts of synthesized P(MAA), P(AAm) and (PAPTMACl) were put into separate beakers and stirred. After 12 h stirring, each hydrogel was separated from solution by centrifugation. The uptake capacity for each metal ion was found and the results are tabulated in Table 2. The results showed that in the competitive experiments, the maximum % *R* of metal ions by the hydrogels occurred in the 0.5 ppm solution of the combined metals. The % *R* for Cu^2+^ and Ni^2+^ was nearly the same and the maximum in all hydrogels. At a high concentration of up to 8 ppm, the maximum % *R* was obtained for Zn^2+^ due to the larger ionic radius of Zn compared to that of the other heavy metal ions, which prevented their penetration in the hydrogel network.

**Table 2:** Competitive % *R* of heavy metal ions by hydrogels

Salt conc. (ppm)	P(MAA) % *R*				P(AAm) % *R*				P(APTMACl) % *R*			
	Cu	Co	Ni	Zn	Cu	Co	Ni	Zn	Cu	Co	Ni	Zn
0.5	100	83	100	73	88	55	46	65	100	77	100	38
5	96	82	91	98	50	51	67	93	46	75	58	83
8	96	62	92	94	69	40	72	78	68	45	62	98

### Absorption isotherms

**Fig. 6:**
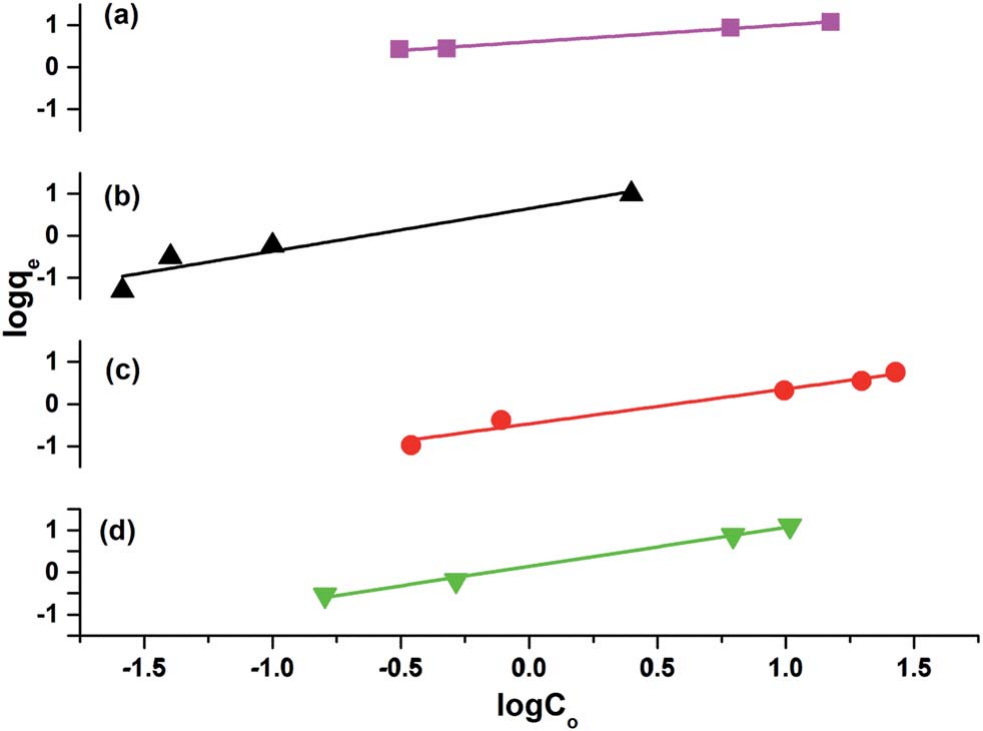
Freundlich isotherms for (a) P(MAA)–Co (b) P(MAA)–Ni (c) P(APTMACl)–Cu and (d) P(AAm)–Ni.

The results attained from the uptake of heavy metal ions by the hydrogels were utilized for the determination of the nature of the absorption process by applying Freundlich and Langmuir isotherms. Fig. 6, in which log *q*_e_*vs.* log *C*_e_ is plotted (eqn (3)), illustrates the general plot of the Freundlich isotherms. The constants were obtained from the intercept and slope of the linear trends.3
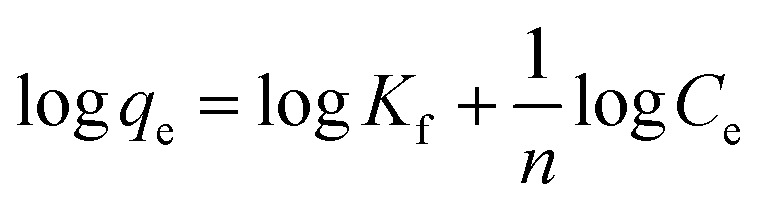
where *q*_e_ is the amount in mg g^−1^ of absorbent absorbed per gram of dried hydrogels at the equilibrium state of absorption, *C*_e_ is the concentration of the absorbate solution at the equilibrium state in ppm, *K*_F_ is the equilibrium constant related to the capacity of absorption and *n* is related to the absorption intensity.

The Langmuir absorption isotherm was also applied by plotting *C*_e_/*q*_e_*vs. C*_e_ (eqn (4)) to find the absorption process.4
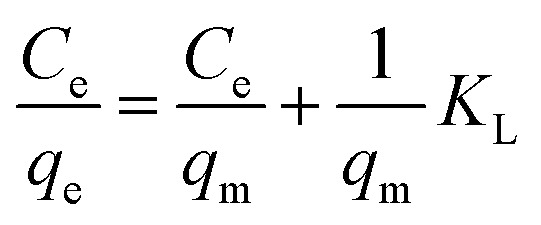
where *C*_e_ is the concentration of the absorbate at equilibrium in ppm, *q*_e_ is the amount in mg g^−1^ of absorbent absorbed per gram of dried hydrogels at the equilibrium state of absorption, *q*_m_ (mg g^−1^) is the maximum amount of absorbent absorbed per gram of hydrogel and *K*_L_ is the equilibrium constant for the Langmuir absorption.

The values of *R*^2^ given in Table 3 for the Freundlich isotherm are nearer to 1 and show a more linear pattern as compared to those of the Langmuir isotherm. All metal ions follow the Freundlich isotherm except Cu^2+^ with P(MAA), which followed the Langmuir absorption isotherm as shown in Fig. S4.† This confirms that Cu has a greater ability to form complexes as compared to the rest of the metals, and was preferentially absorbed chemically.

**Table 3:** Kinetic parameters calculated from different isotherms applied for the uptake study of heavy metal ions

Hydrogel	Metal ion	Langmuir isotherm constant			Freundlich isotherm constant		
		*K*_L_ (L g^−1^)	*q*_m_	*R* ^2^	*K*_F_ (L g^−1^)	1/*n*	*R* ^2^
P(MAA)	Cu	0.11	19.67	0.98	12.34	0.342	0.90
P(MAA)	Co	−2.16	−20.26	0.081	15.018	1.021	0.99
P(MAA)	Ni	1.003	8.24	0.32	4.470	1.018	0.88
P(MAA)	Zn	−1.54	−2.59	−0.32	9.726	0.982	0.98
P(AAm)	Cu	−23.99	−1.29	−0.002	0.143	1.433	0.87
P(AAm)	Co	45.33	65.63	0.003	1.381	0.967	0.99
P(AAm)	Ni	9.04	11.03	0.88	0.965	0.926	0.99
P(AAm)	Zn	−5.54	−4.30	0.17	0.388	1.008	0.82
P(APTMACl)	Cu	30.38	10.46	0.48	0.340	0.830	0.96
P(APTMACl)	Co	2.72	11.50	0.74	1.209	0.336	0.92
P(APTMACl)	Ni	3.28	4.93	0.98	0.962	0.597	0.99
P(APTMACl)	Zn	−87.34	−67.15	−0.40	4.018	0.404	0.96

### Catalytic performance of hybrid hydrogels

Dyes are highly toxic, causing heart problems, vomiting, tissue rupture, jaundice, shudders and Heinz body formation in humans,^31,32^ and similarly to dyes, nitroarenes are also highly toxic but can cause drowsiness, cyanosis, confusion, headaches, irritation of the eyes, misperception, giddiness, queasiness, paraesthesia, methemoglobinemia, *etc.*^33^ The degradation or reduction of both dyes and nitroarenes to fruitful products in order to protect the environment from their worst effects is very important. These pollutants are selected and reduced to essential products for the pharmaceutical, pigment and various other industries using hybrid hydrogels as catalytic systems in aqueous media.

The prepared P(MAA)–M, P(AAm)–M and P(APTMACl)–M (where M = Cu, Co and Ni) hybrid hydrogels were used as catalysts for the reduction of dyes (MB, MO and CR) and nitroarenes (4-NP and NB).

The catalytic activity of the hybrid hydrogels for the reduction and degradation of nitroarenes and azo dyes was monitored using UV-vis absorption spectroscopy. Solutions of the MB, CR and MO dyes appeared blue, red and orange in colour, respectively. 4 mL of each dye (MB, MO and CR) solution was poured into separate quartz cuvettes, and the UV spectra were measured. It was found that for MB, *λ*_max_ occurs at 660 nm, for MO *λ*_max_ is 484 nm and for CR *λ*_max_ is observed at 497 nm due to the allowed π–π* transition.

For the nitroarenes (4-NP and NB), 30 μL of each prepared nitroarene was added to separate quartz cuvettes, and into these 3 mL of deionized water was added. The color of the solution appeared to be light yellow for 4-NP and colorless for NB. The UV peak appeared at *λ*_max_ = 317 nm for 4-NP and for the NB solution the recorded peak for *λ*_max_ was at 285 nm.

It was observed that without catalysts no reduction took place, even in the presence of a large amount of reducing agent. Although, thermodynamically, reduction is feasible, it is kinetically restricted due to the large potential difference between the electron donor and acceptor, which results in a high energy barrier. With a small amount of a catalyst, reduction takes place.^34,35^

**Fig. 7:**
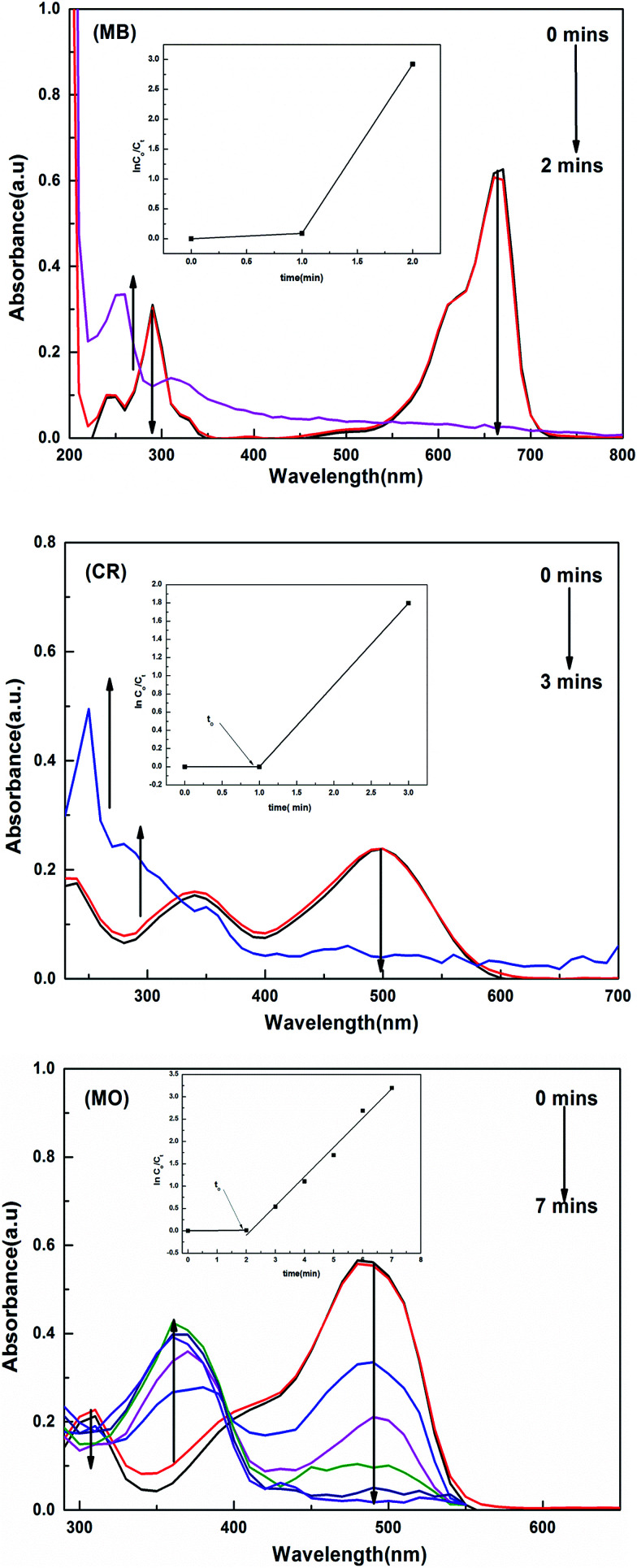
UV/vis spectra and *k*_app_ plots (inset) for the reduction process catalysed by P(MAA)–Cu.

When a certain amount of a hybrid hydrogel was added to the dye solution containing a high amount of the reducing agent, the BH_4_^−^ ions and reactants (MB, MO and CR) are adsorbed on the surface of the metal nanoparticles, and the transfer of electrons takes place from the borate ions to the surface of the metal particles and then to the reactants, converting them to the reduced form, which was physically observed by the disappearance of their colors. The UV-visible spectra collected for the reduction of MB, CR and MO are given in Fig. 7.

The reduction rate constant was calculated by measuring the decrease in intensity of the absorption peaks with time. During the reduction process, the amounts of reducing agent and catalyst used were kept constant and the reaction was carried out with an excess amount of NaBH_4_. The reaction was supposed to follow pseudo first order kinetics. By plotting ln(*C*_o_/*C*_*t*_) *vs.* ‘*t*’, the value of *k*_app_ (apparent rate constant) was calculated from the slope, where *C*_o_ is the initial and *C*_*t*_ is the final concentration of the dye solution at any time *t*.

The *k*_app_ values were obtained for all catalysts and are tabulated in Table 4. The results indicate that the catalytic activity of the P(MAA) hydrogels with a metal (Cu, Co and Ni) loaded is higher, as compared to the P(AAm) and P(APTMACl) hybrid hydrogels. This was due to the fact that the –COOH groups of the P(MAA) hydrogel were converted into the –COO^−^ form in the aqueous medium, and became capable of entrapping a higher amount of metal ions, and after conversion into nanoparticles, the electrostatic forces of repulsion in the polymer network provide stability to these particles. In terms of the hydrogels, the second highest catalytic activity was observed for the P(APTMACl) hybrid hydrogels, although in this case there were fewer entrapped metal ions, but after reduction the metal particles were stable due to electrostatic repulsion. The lowest catalytic activity and stability was found for the P(AAm) hybrid hydrogels, because here only hydrophilic interactions are responsible for keeping the metal ions and particles inside the network. Furthermore, in the P(MAA) hybrid hydrogels, the highest catalytic activity was attributed to Cu. This was due to the easy transfer of electrons from the surface of Cu, as the number of unpaired electrons is lower and hence the nuclear forces of attraction in the case of Cu was high; these properties enhance the catalytic activity of Cu.^29^ The observed *k*_app_ values for the reduction of MB, MO and CR catalysed by P(MAA)–Cu in the present study were ∼2.8, 1.9 and 1.3 min^−1^, respectively, which are very high as compared to those in previous literature.^36^

**Table 4:** The calculated *k*_app_ values for the hybrid hydrogels used as catalysts in the reduction of dyes

Dye	*k*_app_ (min^−1^) P(MAA)–			*k*_app_ (min^−1^) P(AAm)–			*k*_app_ (min^−1^) P(APTMACl)–		
	Cu	Co	Ni	Cu	Co	Ni	Cu	Co	Ni
MB	2.8316	0.6267	0.7246	1.6409	0.3264	0.5371	1.9018	0.3103	0.5505
MO	1.9022	0.3518	0.6793	0.6557	0.3147	0.4333	0.9201	0.2932	0.5527
CR	1.3020	0.2465	0.5124	0.3996	0.1047	0.1057	0.156	0.0598	0.1092

It was also observed that the value of *k*_app_ for CR was lower in comparison to that of MB and MO, irrespective of the catalyst applied. The main reason for this is the larger structure of CR, which occupies a greater area on the surface of the catalyst. In MO, only one azo (–N=N–) group is present, while CR has two such groups, and for this reason all catalysts used for CR have a lower value of *k*_app_.

**Fig. 8:**
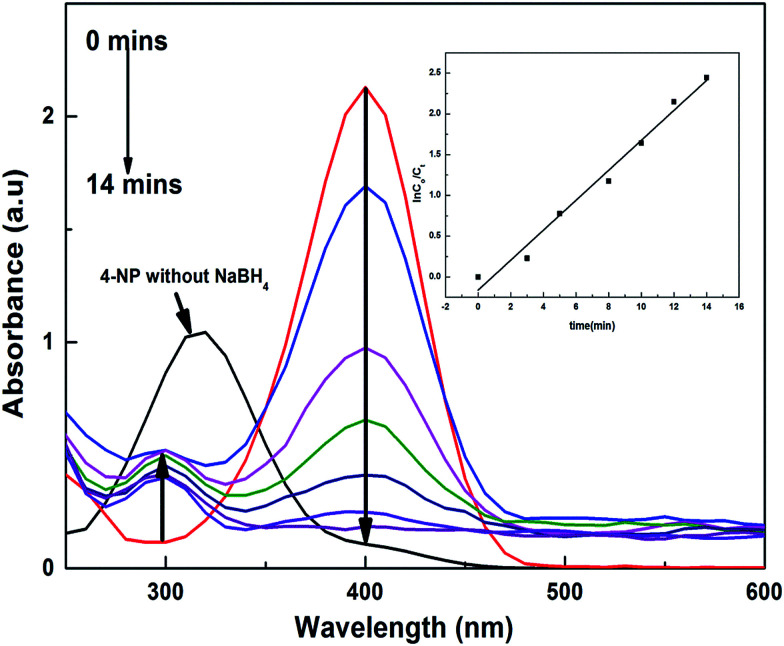
UV/vis spectra and linear plot (inset) for the 4-NP reduction process by P(AAm)–Ni.

For the 4-nitrophenolate ions (4-NP^−^), *λ*_max_ is at 400 nm because of the low energy π–π* symmetric excitation, and for NB *λ*_max_ is at 267 nm because of n–σ* excitation. The solution of 4-NP gives a peak of *λ*_max_ at 317 nm, and when a certain amount of NaBH_4_ was added to this solution, it generated 4-NP^−^, which shifts the peak to 400 nm; with addition of a catalyst, the reactants accumulated on the surface of the catalyst and favoured the transfer of electrons from borohydride to the targeted reactants, which were reduced into benign products, giving new peaks in the UV-visible spectral range, as shown in Fig. 8. The *k*_app_ values for each catalyst are tabulated in Table 5, which shows that the *k*_app_ values for the P(APTMACl) hybrid hydrogels were found to be higher for 4-NP as compared to those for other catalysts. This is because of the positive charge on the P(APTMACl) hydrogel network, so due to the electrostatic forces of attraction the availability of 4-NP^−^ at the surface site was greater and favours the rapid reduction process. From Table 5, it was also concluded that the values of *k*_app_ for the reduction of NB for all synthesized catalysts were lower than the values of *k*_app_ for 4-NP, which correlates with the high stability of 4-NP compared to NB.

**Table 5:** The *k*_app_ for the hybrid hydrogels used as catalysts in the reduction of nitroarenes

Nitroarene	*k*_app_ (min^−1^) P(MAA)–			*k*_app_ (min^−1^) P(AAm)–			*k*_app_ (min^−1^) P(APTMACl)–		
	Cu	Co	Ni	Cu	Co	Ni	Cu	Co	Ni
4-NP	0.9095	0.3410	0.3663	0.7988	0.1273	0.1836	0.7866	0.668	0.6719
NB	0.3647	0.1396	0.3341	0.3597	0.25219	0.2612	0.3345	0.3230	0.3291

Żelechowska *et al.*^37^ and Nanda *et al.*^38^ used highly expensive materials for the generation of Cu and Au based catalysts, respectively, and applied these for 4-NP reduction. The *k*_app_ values obtained, 0.007 s^−1^ and 0.1057 min^−1^, are much less than the value of 0.9095 min^−1^ obtained in this study.

### Stability and recycling activity of prepared catalysts

**Fig. 9:**
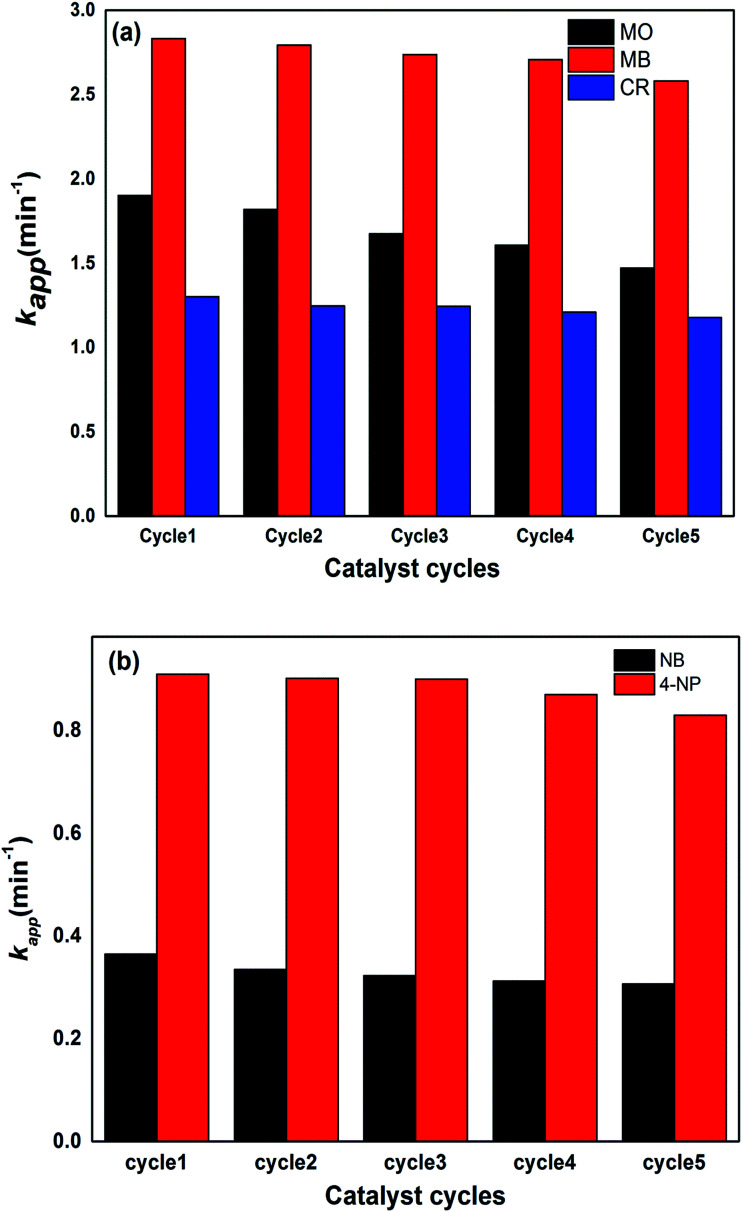
Catalytic reduction of (a) dyes and (b) nitroarenes using P(MAA)–Cu as the catalyst.

For the stability and recycling of the prepared hybrid hydrogel catalysts, experiments were performed that showed that the prepared catalysts were stable for a long time because no changes in catalytic activities were observed after one month since their synthesis. The recycling capacity was checked by carrying out five consecutive reduction cycles with a negligible decrease in their catalytic activities. After each cycle, the catalyst was separated from the reaction mixture through filtration, washed with deionized water, dried and used again in the next cycle. The results of five consecutive cycles for the catalytic reduction of dyes and nitroarenes with P(MAA)–Cu are shown in Fig. 9a and b.

The % *R* was found to show a decrease from 94 to 91% for CR, MB and MO, and 92 to 89% for 4-NP and NB, from the first to the fifth cycle of the reduction process.

From the aforementioned results, it is clear that the % *R* efficiency for MB with P(MAA)–Cu was high as compared to that for CR and MO, because of the favourable strong electrostatic interactions of anionic hydrogels with cationic dyes, due to which reactants were adsorbed more easily on the surface of the catalysts and hence were transformed into products. The % *R* values of all of the dyes for five cycles were nearly equal, with slight differences which were due to leakage of the catalyst during the recovery and washing process. All of the prepared hybrid hydrogel catalysts were proven to be the most effective catalysts, in terms of their stability and recycling capacity. This proves their applicability for the removal of nitroarenes and dyes from manufacturing contaminants.

## Conclusion

Herein, the preparation of P(MAA), P(AAm) and P(APTMACl) hydrogels *via* free radical polymerization at room temperature was demonstrated. The prepared hydrogels were successfully used as sorbents for the removal of heavy metal ions *i.e.* Cu^2+^, Co^2+^, Ni^2+^ and Zn^2+^. The metal ion absorbed hydrogels underwent *in situ* reduction using NaBH_4_ for the generation of hybrid hydrogels. The amounts of metal ions absorbed by the hydrogels were determined using flame AAS and the percent of metal content was determined using TGA. P(MAA)–M, P(AAm)–M and P(APTMACl)–M (where M = Cu, Co and Ni) hybrid hydrogels were used as catalysts for the reduction of macropollutants *i.e.* MB, MO and CR and micropollutants *i.e.* 4-NP and NB. Excellent catalytic activity was attained for all catalysts during the reduction reactions. For the nature and mechanism of metal ion absorption, two isotherms, Freundlich and Langmuir, were applied to the absorption data, and it was confirmed that absorption of all of the metal ions follows the Freundlich absorption model, except P(MAA)–Cu, which obeys the Langmuir absorption because of its complexing properties. Among all of the catalysts, the P(MAA)–Cu hybrid hydrogel showed superior catalytic activity. The catalysts were found to be stable for a long time and can be reused in multiple cycles without reduction in their catalytic activity. Their absorbent and catalytic activities can be extended to other toxic pollutants.

## Conflicts of interest

I, on the behalf of all of the co-authors, confirm that we have no conflict of interest in submission of this paper to RSC Advances.

## Supplementary Material

RA-008-C8RA00578H-s001
