# Supplementary material for: Uptake of heavy metal ions from aqueous media by hydrogels and their conversion to nanoparticles for generation of a catalyst system: two-fold application study
Source: RSC Adv. 2018 Apr 18;8(27):14787–97. doi: 10.1039/c8ra00578h (PMC9079941; doi:10.1039/c8ra00578h)
Supplement: RA-008-C8RA00578H-s001 [file RA-008-C8RA00578H-s001.pdf]

Electronic Supplementary Material (ESI) for RSC Advances.
This journal is © The Royal Society of Chemistry 2018

Uptake of heavy metal ions from aqueous medium by hydrogels and its conversion to nanoparticles for generation of catalyst system: Two fold application study

Rida Javed, Luqman Ali Shah*, Murtaza Sayed, Muhammad Saleem Khan

National Centre of Excellence in Physical Chemistry, University of Peshawar, Peshawar 25120 Pakistan

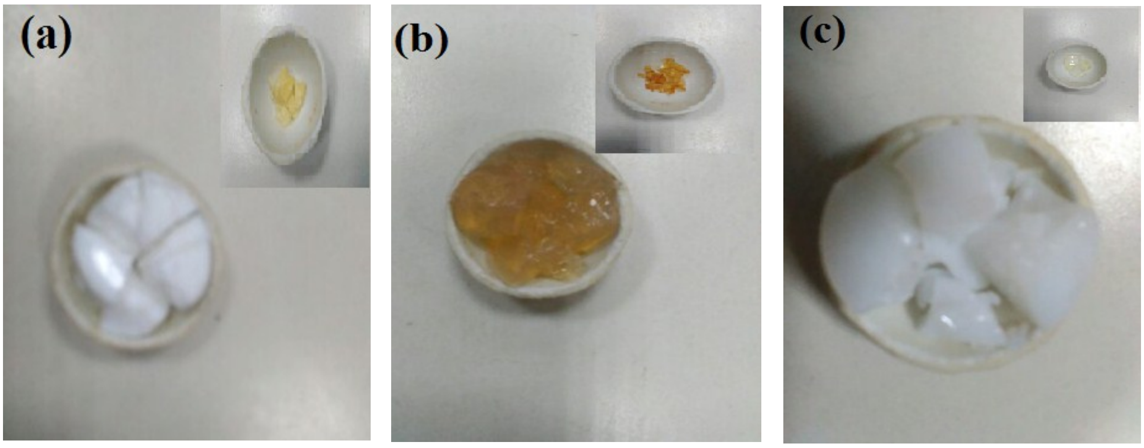

Fig. S1 Physical appearance of synthesized wet (a) P(MAA) (b) P(APTMACl) and (c) P(AAm) and dried (insets) hydrogels

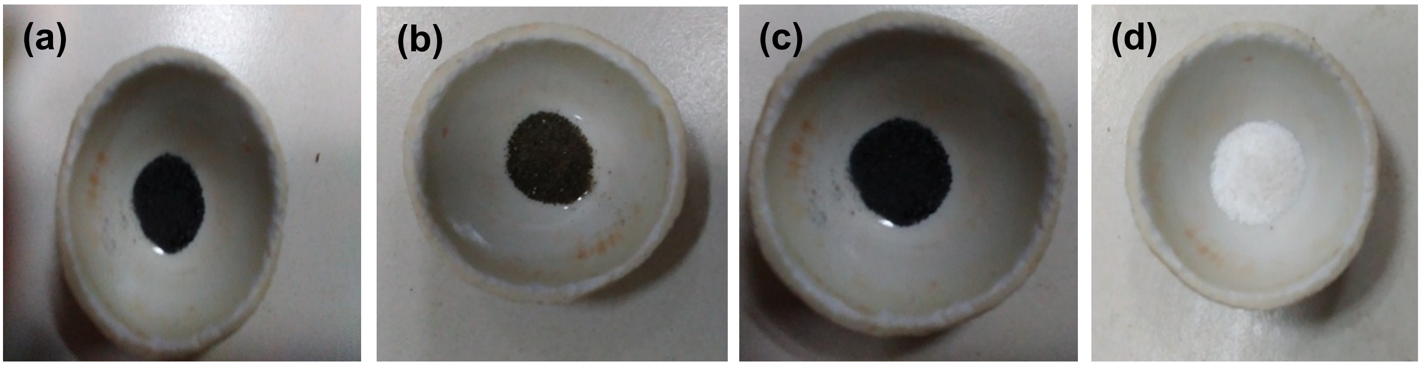

Fig. S2 Physical appearance of prepared (a) P(MAA)-Cu (b) P(MAA)-Co (c) P(MAA)-Ni and (d) P(MAA)-Zn hybrid hydrogels

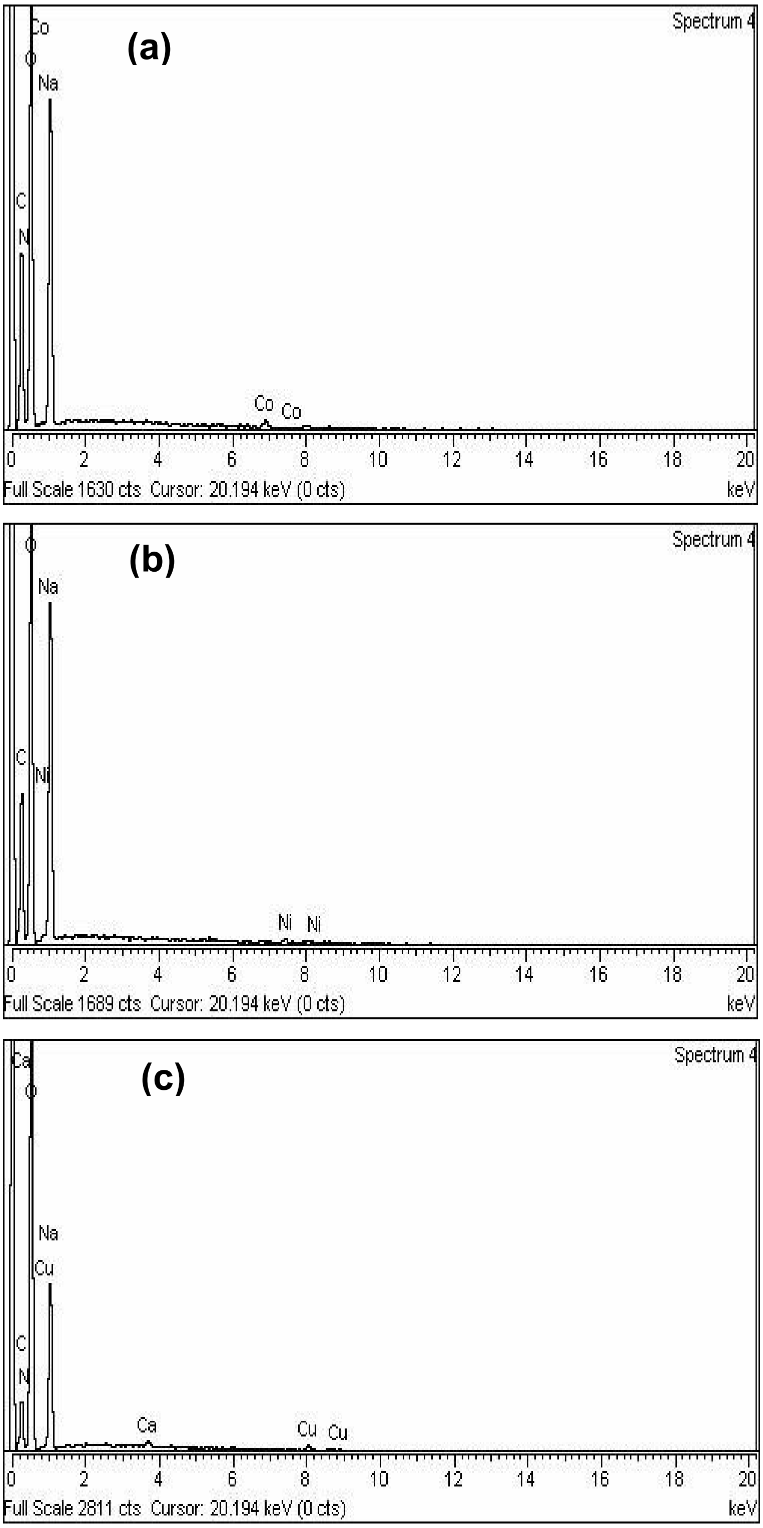

Fig. S3 EDX spectral analysis for (a) P(MAA)-Co (b) P(MAA)-Ni and (c) P(MAA)-Cu

Table S1 Temperature, wt. loss and amount of metals for pure and hybrid P(MAA) hydrogels

**Sample**	**Ist(degradation)**		**2^{nd}(degradation)**		**3^{rd}(degradation)**		**Metal content (%)**
	**Temp (°C)**	**wt. loss (%)**	**Temp (°C)**	**wt. loss (%)**	**Temp (°C)**	**wt. loss (%)**	
P(MAA)	58-327	33%	343-449	60%	No	No	0
P(MAA)-Cu	42-134	26%	406-520	28%	=	=	26
P(MAA)-Co	34-155	26%	407-501	21%	659-750	8%	25
P(MAA)-Ni	35-133	24%	400-488	21%	643-749	9%	25
P(MAA)-Zn	39-114	22%	405-510	26%	640-750	8%	26

Table S2 Temperature, wt. loss and metal content for P(APTMACl) and P(AAM) hydrogels

Sample	1st degradation		2nd degradation		Metal content (%)
	Temp (°C)	wt. loss (%)	Temp (°C)	wt. loss (%)	
P(APTMACl)	50-224	21	230-460	61	0
P(APTMACl)-Cu	45-120	16	153-460	45	21
P(APTMACl)-Co	123-300	39	326-460	24	19
P(APTMACl)-Ni	72-240	37	243-460	26	19
P(APTMACl)-Zn	50-207	23	213-460	36	24
P(AAm)	70-316	34	336-460	35	0
P(AAm)-Cu	90-303	22	323-460	32	15
P(AAm)-Co	70-330	31	338-460	18	20
P(AAm)-Ni	72-240	15	243-460	37	17
P(AAm)-Zn	160-219	43	228-460	19	3

Table S3 % R capability of hydrogels towards different concentration of metal ions solution

Concentrations	P(MAA) %R				P(AAm) % R				P(APTMACl) % R			
	Cu	Co	Ni	Zn	Cu	Co	Ni	Zn	Cu	Co	Ni	Zn
5 ppm	99	94	98	70	54	67	77	49	62	68	85	45
10 ppm	100	96	97	87	58	68	76	49	63	71	81	53
20 ppm	98	94	86	97	68	78	88	38	49	64	86	29
40 ppm	100	99	100	97	43	71	94	36	33	66	76	28

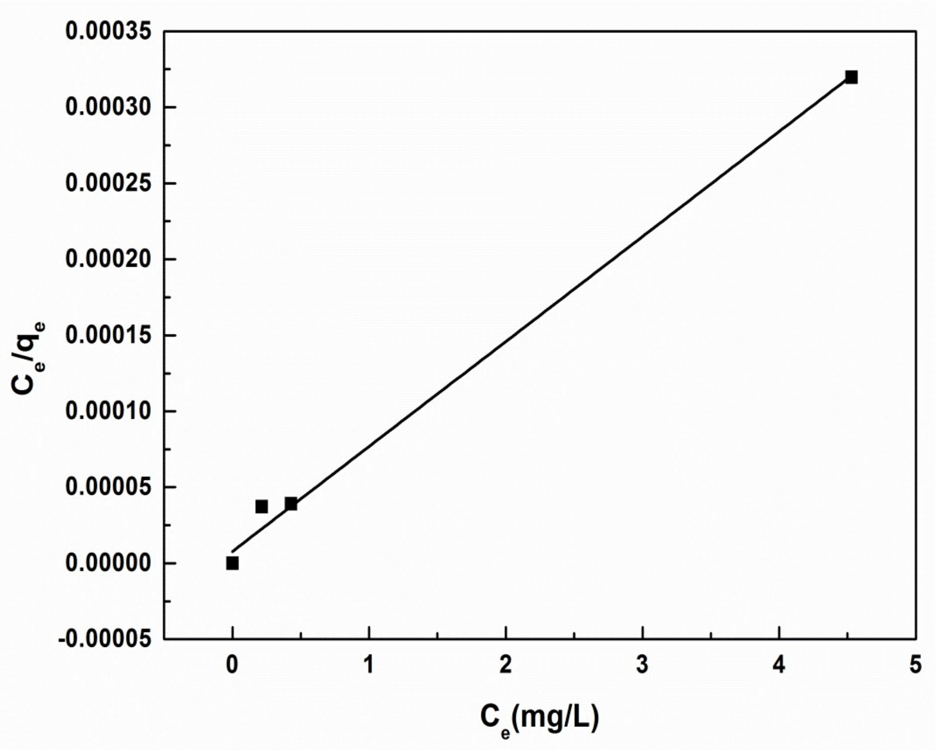

Fig. S4 Langmuir isotherm for absorption of Cu by P(MAA) from aqueous medium
